# Relationship between time to target temperature and outcome in patients treated with therapeutic hypothermia after cardiac arrest

**DOI:** 10.1186/cc10116

**Published:** 2011-03-25

**Authors:** Moritz Haugk, Christoph Testori, Fritz Sterz, Maximilian Uranitsch, Michael Holzer, Wilhelm Behringer, Harald Herkner

**Affiliations:** 1 Department of Emergency Medicine, Medical University of Vienna, Währinger Gürtel 18-20/6D, Wien, 1090, Austria

## Abstract

**Introduction:**

Our purpose was to study whether the time to target temperature correlates with neurologic outcome in patients after cardiac arrest with restoration of spontaneous circulation treated with therapeutic mild hypothermia in an academic emergency department.

**Methods:**

Temperature data between April 1995 and June 2008 were collected from 588 patients and analyzed in a retrospective cohort study by observers blinded to outcome. The time needed to achieve an esophageal temperature of less than 34°C was recorded. Survival and neurological outcomes were determined within six months after cardiac arrest.

**Results:**

The median time from restoration of spontaneous circulation to reaching a temperature of less than 34°C was 209 minutes (interquartile range [IQR]: 130-302) in patients with favorable neurological outcomes compared to 158 min (IQR: 101-230) (*P *< 0.01) in patients with unfavorable neurological outcomes. The adjusted odds ratio for a favorable neurological outcome with a longer time to target temperature was 1.86 (95% CI 1.03 to 3.38, *P *= 0.04).

**Conclusions:**

In comatose cardiac arrest patients treated with therapeutic hypothermia after return of spontaneous circulation, a faster decline in body temperature to the 34°C target appears to predict an unfavorable neurologic outcome.

## Introduction

For patients who have been successfully resuscitated after cardiac arrest, therapeutic mild hypothermia increases the rate of a favorable outcome in comparison with standard life support. Randomized controlled trials, however, have not shown evidence of whether the time to target temperature correlates with neurological outcome [1-4]. Registries about the practical use of therapeutic hypothermia have also not found a significant association between the timing of therapeutic hypothermia and final outcome [5-7]. We expected a strong relationship between the time to target temperature (<34°C) and neurological outcome. Furthermore, we hypothesized that earlier achievement of target temperature would not necessarily improve outcome.

## Materials and methods

The study was designed as a single-center retrospective cohort study on temperature data extracted from patients' charts by observers blinded to outcome. The protocol and consent procedures were approved by the ethics committee of the Medical University of Vienna and were performed in accordance with the Declaration of Helsinki. This trial qualified for exception from informed consent requirements for emergency research as outlined in the applicable national laws of Austria. The subjects enrolled were in an immediate life-threatening situation, unconscious, and unable to provide consent for trial enrollment and had a mortality approaching 75%. As cardiac arrest is frequently the first manifestation of cardiovascular disease, there was no way to prospectively identify individuals who would become eligible for this trial. However, investigators attempted to notify family members that the subjects had been enrolled. In the event that a patient became competent during the trial period, he or she was approached as soon as feasible by research personnel for notification of enrollment. Data were collected at the Department of Emergency Medicine of the Medical University in Vienna between April 1995 and June 2008 and included data of reports on the same patients [1,8-12] but did not include data focusing on the details of the time to target temperature (Table 1). Participants were consecutive emergency department patients after cardiac arrest in whom spontaneous circulation had been restored and who were receiving therapeutic hypothermia with intensive care in accordance with a standardized protocol [8,13,14].

**Table 1:** Characteristics of patients in the study

	Time to target temperature			
		
	<120 minutes	120 to 220 minutes	>220 minutes	*P *value
Age, years	56 (45-67)	60 (51-71)	58 (49-66)	0.34
Male	40 (65%)	48 (67%)	59 (82%)	0.05
Height, cm	175 (165-180)	175 (165-180)	177 (171-180)	0.01
Weight, kg	78 (70-85)	80 (71-90)	85 (75-95)	0.002
Cardiac arrest				
Out-of-hospital	60 (97%)	64 (89%)	67 (93%)	0.21
Cardiac etiology	40 (65%)	54 (75%)	57 (79%)	0.15
Witnessed	53 (85%)	66 (92%)	63 (88%)	0.52
Bystander BLS^a^	24 (39%)	20 (28%)	16 (22%)	0.10
1st ECG, VF/VT^b^	34 (55%)	46 (64%)	52 (72%)	0.11
Defibrillations	2 (0-3)	2 (1-5)	2 (1-5)	0.55
Adrenaline, mg	3 (1-4)	2 (1-4)	3 (1-4)	0.58
No-flow^c^, minutes	3 (1-10)	3 (0-7)	5 (1-8)	0.51
Low-flow^d^, minutes	20 (14-27)	16 (10-22)	11 (9-23)	0.02
ROSC to admission, minutes	31 (25-42)	36 (24-54)	39 (26-51)	0.25
ROSC to cool, minutes	58 (29-72)	101 (58-130)	86 (43-130)	0.001
ROSC to awakening^e^, days	5 (3-9)	4 (2-6)	4 (3-7)	0.59
ROSC to discharge, days	27 (17-51)	30 (20-46)	28 (21-45)	0.85
ROSC to death, days	7 (3-14)	6 (3-30)	18 (6-83)	0.05
Best CPC^f^	4 (1-4)	3 (1-4)	1 (1-4)	0.006
Favorable neurologic outcome	23 (37%)	34 (47%)	45 (63%)	0.01
Survivors	30 (48%)	36 (50%)	40 (56%)	0.68

Temperature data between April 1995 and June 2008 were collected from 588 patients. The best cerebral performance category (CPC) achieved within 6 months after cardiac arrest was used; this was not available in 17 patients who died during anesthesia. Data are presented as median (interquartile range) and as number (percentage). The Kruskal-Wallis test for continuous data and chi-square test for categorical data were used to compare variables between the surviving and dead groups and between the favorable and unfavorable neurologic outcome (CPC 1-2 versus 3-4) groups. ^a^Patients who have received basic life support from a bystander. ^b^Cardiac arrest with ventricular fibrillation or nonperfusing ventricular tachycardia as the initial cardiac rhythm on electrocardiogram. ^c^Cardiac arrest with no blood flow. ^d^Interval from the first attempt at resuscitation to restoration of spontaneous circulation (ROSC). ^e^Awakening was defined as response to external stimuli with purposeful movements. ^f^A CPC of 1 indicates good cerebral performance (the patient is alert and has normal cerebral function). A CPC of 2 indicates moderate disability (the patient is alert and has sufficient cerebral function to live independently and work part-time). Such patients might have hemiplegia, seizures, ataxia, dysarthria, dysphasia, or permanent memory loss, or other mental changes. A CPC of 3 indicates severe cerebral disability (the patient is conscious but dependent on others for daily support because of impaired brain function). A CPC of 4 indicates a vegetative state.

Neurologic morbidity (a) and mortality (b) were independently compared with the time to target temperature. Neurologic outcome was measured as a Pittsburgh cerebral performance category (CPC) on a five-category scale [15,16]. Patients with good recovery or moderate disability had sufficient cerebral function to live independently and work at least part-time (best CPC 1-2). The Pittsburgh CPCs were assessed on a regular basis by means of structured patient interviews.

The temperature immediately on admission was measured with an infrared tympanic thermometer (Ototemp LighTouch; Exergen Corporation, Watertown, MA, USA). Continuous temperature measurements were made as soon as possible in the commencement of therapeutic cooling with a bladder-temperature probe (Foley catheter temperature sensor; Smiths Medical, Dublin, OH, USA) and with a general-purpose temperature probe (Mon-a-therm General purpose; Mallinckrodt Medical Inc., now part of Covidien, Hazelwood, MO, USA) inserted in the esophagus. Temperature-monitoring data were extracted from patient charts by two independently operating staff members. Temperature recordings in the tympanum, esophagus, and bladder were registered with regard to when the first measurement after restoration of spontaneous circulation was available: at the start of cooling and at the first temperature recording of less than 34°C and 33°C (Tables 2 and 3). Data on cardiac arrest for individual patients were recorded in a database according to the Utstein style [15,16].

**Table 2:** Temperatures in degrees Celsius in correlation to morbidity and mortality

	Best CPC 1-2 285 (48)	Best CPC 3-4 286 (49)	*P *value	Survivors 284 (48)	Dead 304 (52)	*P *value
T_ty _1st	35.3 (34.6-36)	35.4 (34.5-36.1)	0.92	35.2 (34.4-36)	35.5 (34.7-36.2)	0.05
T_ty _cool start	35.1 (34.1-35.8)	34.5 (33.6-35.7)	0.15	34.6 (33.8-35.4)	34.9 (33.9-35.9)	0.29
						
T_es _1st	35.9 (34.9-36.4)	35.2 (34.2-36)	0.05	35.7 (34.9-36.4)	35.2 (34.5-36.4)	0.32
T_es _cool start	35.6 (34.8-36.2)	35.1 (34.2-35.9)	<0.01	35.2 (34.5-36)	35.5 (34.6-36.1)	0.27
						
T_bl _1st	35.6 (34.9-36.2)	35.3 (34.6-35.9)	0.09	35.4 (34.7-36)	35.5 (34.9-36)	0.98
T_bl _cool start	35.8 (34.9-36.5)	35.4 (34.6-36.1)	<0.01	35.7 (34.6-36.3)	35.6 (34.7-36.3)	0.95

Temperature data between April 1995 and June 2008 were collected from 588 patients. The best cerebral performance category (CPC) achieved within 6 months after cardiac arrest was used; this was not available in 17 patients who died during anesthesia. Data are presented as median (interquartile range). The two-sample Wilcoxon rank-sum (Mann-Whitney) test and Fisher exact test, as appropriate, were used to compare variables in the surviving and dead groups and the favorable and unfavorable neurologic outcome (CPC 1-2 versus 3-4) groups. T_bl_, bladder temperature; T_es_, esophageal temperature; T_ty_, tympanic temperature.

**Table 3:** Minutes to target temperature in correlation to morbidity and mortality

	Best CPC 1-2 285 (48)	Best CPC 3-4 286 (49)	*P *value	Survivors 284 (48)	Dead 304 (52)	*P *value
ROSC to T_ty _1st	29 (29-58)	29 (29-58)	0.23	29 (29-58)	29 (29-58)	0.59
ROSC to CoolStart	86 (43-144)	86 (58-115)	0.44	86 (58-130)	86 (43-130)	0.27
ROSC to T_ty _≤34°C	144 (94-230)	144 (115-245)	0.77	151 (94-187)	130 (115-245)	0.56
ROSC to T_ty _≤33°C	173 (130-230)	173 (122-230)	0.92	180 (130-216)	144 (130-245)	0.94
						
ROSC to T_es _1st	58 (29-58)	43 (29-65)	0.50	50 (29-58)	58 (29-86)	0.28
ROSC to CoolStart	86 (43-144)	86 (58-115)	0.44	86 (58-130)	86 (43-130)	0.27
ROSC toT_es _<34°C	209 (130-302)	158 (101-230)	<0.01	202 (115-259)	158 (101-230)	0.57
ROSC to T_es _<33°C	274 (202-389)	245 (144-374)	0.02	259 (166-367)	259 (158-389)	0.68
						
ROSC to T_bl _1st	58 (29-86)	58 (29-72)	0.29	58 (29-72)	58 (29-86)	0.62
ROSC to CoolStart	86 (43-144)	86 (58-115)	0.44	86 (58-130)	86 (43-130)	0.27
ROSC to T_bl _<34°C	230 (158-360)	194 (115-288)	<0.01	216 (144-288)	216 (144-346)	0.73
ROSC to T_bl _<33°C	346 (245-504)	288 (187-418)	<0.01	331 (245-475)	317 (216-446)	0.43
						
CoolStart to T_ty _<34°C	29 (0-43)	14 (0-115)	0.95	14 (0-43)	29 (0-115)	0.42
CoolStart to T_ty _<33°C	29 (14-72)	50 (14-137)	0.17	36 (14-101)	58 (29-130)	0.35
						
CoolStart to T_es _<34°C	86 (43-144)	58 (29-115)	0.01	72 (43-130)	72 (29-144)	0.72
CoolStart to T_es _<33°C	173 (86-274)	115 (58-245)	0.03	137 (72-245)	144 (72-245)	0.32
						
CoolStart to T_bl _<34°C	115 (72-216)	86 (43-187)	<0.01	101 (58-202)	101 (58-216)	0.98
CoolStart to T_bl _<33°C	245 (144-418)	187 (101-317)	0.01	216 (115-389)	202 (101-331)	0.31

Temperature data between April 1995 and June 2008 were collected from 588 patients. The best cerebral performance category (CPC) achieved within 6 months after cardiac arrest was used; this was not available in 17 patients who died during anesthesia. Data are presented as median (interquartile range). The two-sample Wilcoxon rank-sum (Mann-Whitney) test was used to compare variables in the surviving and dead groups and the favorable and unfavorable neurologic outcome (CPC 1-2 versus 3-4) groups. ROSC, restoration of spontaneous circulation; T_bl_, bladder temperature; T_es_, esophageal temperature; T_ty_, tympanic temperature.

The exposure of interest was the time needed from restoration of spontaneous circulation to achieve a temperature of less than 34°C. Esophageal temperature was chosen because it is considered to be more reliable than urinary bladder or tympanic temperature in reflecting core body temperature [17,18]. The primary outcome was a favorable neurological outcome, defined as best CPC 1 (good recovery) or 2 (moderate disability) within 6 months after cardiac arrest. The secondary outcome was all-cause mortality within 6 months of arrest.

### Statistical analysis

We reported continuous variables as medians and 25% to 75% interquartile ranges (IQRs) because they were not generally normally distributed. Categorical variables are reported as counts and percentages. The Mann-Whitney *U *test was used to test the null hypothesis of no difference in time to target temperature. To estimate the size of this effect, we used logistic regression analysis, and favorable neurological outcome was the dependent variable. We categorized the predictor time to target temperature into tertiles of time from restoration of spontaneous circulation to esophageal temperature of not more than 34°C (<120, 120 to 220, and >220 minutes) because the assumption of a linear effect did not hold when used as a continuous variable despite standard transformations. Potential confounders were identified by inspection after tabulating them along with exposure and outcome. For formal hypothesis testing, we used the Mann-Whitney *U *test, the Fisher exact test, or the Pearson chi-square test as appropriate (Tables 1, 2, 3). To adjust the effect of time to target temperature on neurological outcome to potential confounders, we developed a multivariable logistic regression model. For covariates, we included factors that were associated with exposure as well as with outcome in our bivariate analyses but were not considered to be part of the causal pathway from exposure to the outcome. These factors included age, total amount of adrenaline needed to achieve restoration of spontaneous circulation, temperature at beginning of cooling, cardiac arrest with ventricular fibrillation, and nonperfusing ventricular tachycardia as the initial cardiac rhythm (Table 4). Time to restoration of spontaneous circulation, no-flow time, and adrenaline dose were closely correlated to each other, so we included only adrenaline dose as the most robust variable in the model to cope with co-linearity. The likelihood ratio test was used to assess deviations from linearity and first-order interactions. The Hosmer-Lemeshow goodness-of-fit statistic indicated a poor fit if the resulting significance value was less than 0.05. We calculated bootstrapped 95% confidence intervals. This procedure was repeated for overall survival as the secondary outcome. Stata software (version 8.1; StataCorp LP, College Station, TX, USA) was used to analyze the data. A two-sided *P *value of less than 0.05 was generally considered to be statistically significant.

**Table 4:** Multivariable logistic regression for favorable outcome

	Odds ratio (bootstrapped 95% confidence intervals)	*P *value
Time to target temperature^a ^- crude	1.69 (1.22-2.34)	0.002
Time to target temperature^a,b^	1.86 (1.03-3.38)	0.04
Time to target temperature adjusted for		
Age, years	0.95 (0.92-0.99)	0.01
Epinephrine, mg^c^	0.70 (0.46-1.04)	0.08
T_bl _cool start, °C^d^	1.08 (0.71-1.63)	0.72
1st ECG, VF^e^	2.54 (0.84-7.71)	0.10

^a^Linear effect for tertiles of time from restoration of spontaneous circulation (ROSC) to esophageal temperature of not more than 34°C (<120, 120 to 220, and >220 minutes). ^b^Hosmer-Lemeshow chi-squared = 2.67, *P *= 0.85, indicating good model fit. Temperature data between April 1995 and June 2008 were collected from 588 patients. ^c^Total amount of epinephrine in milligrams needed to achieve ROSC. ^d^Bladder temperature in degrees Celsius at beginning of cooling. ^e^Cardiac arrest with ventricular fibrillation or nonperfusing ventricular tachycardia as the initial cardiac rhythm on electrocardiogram.

## Results

A total of 2,536 cardiac arrest patients were assessed; 1,948 of these patients were not cooled. Thus, 588 patients following the treatment algorithm were studied; they included (a) a group of 285 (48%) patients with favorable outcomes and (b) a group of 284 (48%) survivors (Tables 1, 2, 3). All patients included in the analysis were available for follow-up neurologic status evaluation; 17 patients died during sedation, analgesia, and paralysis. At baseline, the patients in the group comparing morbidity were generally similar to the group comparing mortality (Table 1). The methods and strategies used for cooling were not different between groups.

**Figure 1:**
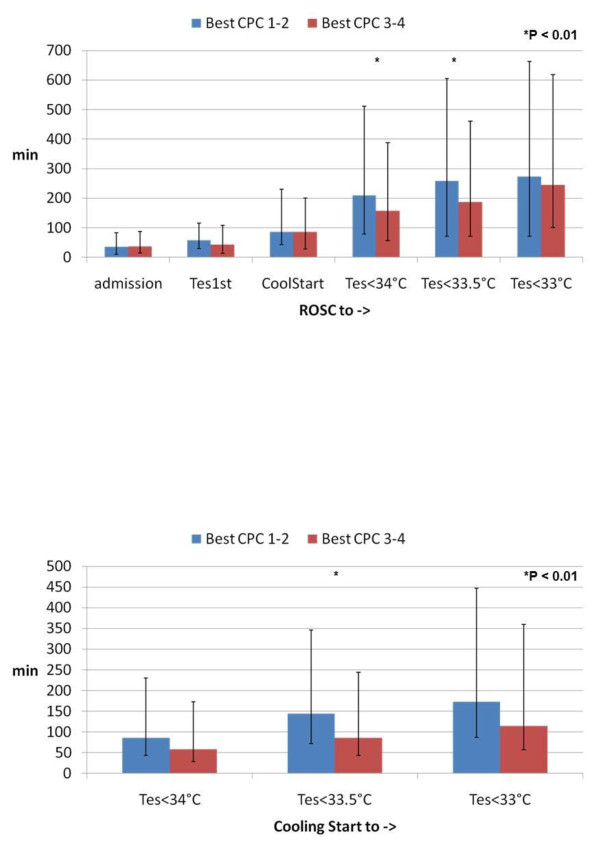
**Time to Target Temperature (T2TT) correlated to the best cerebral perforemance catergory (CPC)**. Temperature data between April 1995 and June 2008 were collected from 588 patients. The best cerebral performance category achieved within 6 months after cardiac arrest was used. A CPC of 1 indicates good cerebral performance (the patient is alert and has normal cerebral function). A CPC of 2 indicates moderate disability (the patient is alert and has sufficient cerebral function to live independently and work part-time). Such patients might have hemiplegia, seizures, ataxia, dysarthria, dysphasia or permanent memory loss, or other mental changes. A CPC of 3 indicates severe cerebral disability (the patient is conscious but dependent on others for daily support because of impaired brain function). A CPC of 4 indicates a vegetative state. Time is expressed in minutes from restoration of spontaneous circulation (ROSC) to hospital admission (admission), to first monitored esophageal temperature (T_es _1st), to start of cooling (Cool Start), and to esophageal temperature (T_es_) of less than 34°C, 33.5°C, and 33°C. Data are presented as median and interquaritle range from the 25th to the 75th percentile.

In all patients, more initial temperature recordings were available from the tympanum (*n *= 313) than from the esophagus (*n *= 102) or bladder (*n *= 179), and there were no differences between groups (Table 2). The time from restoration of spontaneous circulation until the temperature recordings at cooling start in the tympanum (*n *= 112), esophagus (*n *= 177), and bladder (*n *= 276) did not differ significantly between the groups (Table 2 and Figure 1). Time variables are presented in Table 3.

We found 86% higher odds of a favorable neurological outcome with an increase in each tertile of time to target temperature (adjusted odds ratio 1.86, 95% confidence interval 1.03 to 3.38; *P *= 0.04) (Table 4). The median time needed to achieve an esophageal temperature of less than 34°C was not associated with overall survival (202 minutes, 25% to 75% IQR 115 to 259 for survivors versus 158 minutes, 25% to 75% IQR 101 to 230 for nonsurvivors) (Table 3). Cooling methods used are shown in Table 5.

**Table 5:** Cooling methods in correlation to morbidity and mortality

	Survivors 284 (48)	Dead 304 (52)	*P *value	Best CPC 1-2 285 (48)	Best CPC 3-4 286 (49)	*P *value
Cooling method						
Endovascular	60 (21)	58 (19)	0.60	65 (23)	53 (17)	0.56
Head	10 (4)	7 (2)	0.54	6 (2)	11 (4)	0.13
Surface ice	43 (15)	36 (12)	0.62	29 (10)	50 (17)	0.01
Nasopharyngeal	7 (2)	7 (2)	0.95	5 (2)	9 (3)	0.18
IV	10 (4)	10 (3)	0.94	8 (3)	12 (4)	0.22
IV + endovascular	51 (18)	55 (18)	0.68	52 (18)	54 (18)	0.38
IV + surface air	5 (2)	9 (3)	0.29	5 (2)	9 (3)	0.18
IV + surface ice	6 (2)	8 (3)	0.57	6 (2)	8 (3)	0.39
IV + surface water	5 (2)	8 (3)	0.40	11 (4)	2 (1)	0.06
Surface air	39 (14)	65 (21)	0.05	53 (19)	51 (17)	0.54
Surface water	30 (11)	27 (9)	0.82	24 (8)	33 (11)	0.11
Surface waterbath	8 (3)	2 (1)	0.15	8 (3)	2 (1)	0.15
Other or mixed	13 (5)	9 (3)	0.48	13 (5)	9 (3)	0.73
						
Invasive cooling	155 (55)	160 (53)		161 (56)	154 (51)	
Noninvasive cooling	129 (45)	144 (47)	0.68	124 (44)	149 (49)	0.19
						
Out-of-hospital cooling	25 (9)	23 (8)		19 (7)	29 (10)	
In-hospital cooling	259 (91)	281 (92)	0.65	266 (93)	274 (90)	0.23
						
No IV cooling	207 (73)	214 (70)		203 (71)	218 (72)	
IV cooling alone	10 (4)	10 (3)		8 (3)	12 (4)	
IV+ cooling	67 (24)	80 (26)	0.75	74 (26)	73 (24)	0.68

Temperature data between April 1995 and June 2008 were collected from 588 patients. Data are presented as number (percentage). Fisher exact test, Pearson chi-square test, and Wald test for comparing the cooling methods with 'endovascular' as reference were used to compare variables, such as favorable and unfavorable neurologic outcome (cerebral performance category [CPC] 1-2 versus 3-4) groups, in the survivors and dead. IV, intravenous.

## Discussion

Our results show a strong relationship between the time to target temperature and neurological outcome among comatose patients in whom spontaneous circulation had been restored after cardiac arrest and therapeutic hypothermia was induced. A shorter time until systemic cooling to an esophageal temperature below 34°C seems to be able to predict unfavorable neurologic outcome. If faster cooling is detrimental or patients with more severe neurological damage show a faster cooling rate has to be further evaluated.

This study has the limitations of a retrospective, observational, and descriptive single-center registry, but the observers of temperature data were blinded to outcome. All eligible patients may not have been cooled, and many factors in regard to patient heat transfer are not available, particularly in the beginning of post-arrest cooling, which lies in the nature of implementing a new therapeutic strategy. Even if a selection bias exists, the time to target temperature over the years remains a factor independent from any bias. To analyze the effect of the time to target temperature on morbidity and mortality, the cohort was evaluated independently in regard to the best-ever-achieved CPC during the follow-up period and in regard to survival. Therefore, it was necessary to exclude the patients dying during sedation, analgesia, and paralysis from the morbidity evaluation.

Though similar to those in other hypothermia trials, the median time intervals in our study were still lengthy. It took more than 80 minutes to commence cooling therapy and around 3 hours to achieve the target temperature. The time to initiation of cooling was not different between groups, showing that it does not correlate with the time to target temperature. Therefore, it appears that patients with a worse responsiveness to cooling are those with a better outcome; however, this cannot be proven without measuring heat transfer in a prospective trial. Along the same lines, the data show that esophageal temperatures at the beginning of cooling were half a degree lower in patients with unfavorable neurologic outcomes in comparison with those with favorable neurologic outcomes. Thus, patients with unfavorable neurologic outcomes could reach target temperature more quickly. The delay in time to target temperature could reflect a stronger natural homeostatic central-nervous physiological response in those with better outcomes; this might also explain the association with improved outcome but not survival.

The standard for temperature monitoring in our patients after cardiac arrest is to get the first temperature at the tympanum and thereafter as soon as possible in the esophagus and bladder. By presenting the temperature data from the three different sites separately, we want to show that our findings are independently the same (Table 2). This and the long time intervals to initiation of cooling and achieving the target temperature justify basing the calculations only on the ones having an esophageal temperature probe and using the initial bladder temperature for the multivariate analysis. Even if temperatures at the beginning of cooling were lower in patients with unfavorable neurologic outcomes, the difference remains with 0.4°C marginal (Table 2) and we have controlled for that variable and other confounders in our adjusted analysis (Table 3). However, many known and possibly still unknown confounders have not been taken care of and therefore our results can be used only for hypothesis generating of future trials necessary to answer this and many other questions related to therapeutic hypothermia. In addition, the time to target temperature from start of cooling, reflecting the cooling rate, proved to be shorter in the group with unfavorable neurologic outcomes. The reason for this surprising result might be of an associative nature and could be explained by the compromised thermoregulation with cerebral insults [12,19]. The data are important for those planning future prospective studies of early versus delayed cooling.

The findings of the present study appear to be contradictory to the notion that achieving mild therapeutic hypothermia earlier and faster helps to reduce hypoxic brain injury and favors a positive neurologic outcome [3,20,21]. It is disappointing that no additional clinical data of patients with cardiac arrest and cooling help to explain that discrepancy. Therefore, all discussions of our findings remain speculative and will not be able to answer whether rapid cooling will cause more hemodynamic changes or other cerebral effects that alter neurologic outcome. Alternatively, it appears that sicker patients lack homeostatic mechanisms to maintain normothermia and therefore cool faster. Unfortunately, the question of how soon cooling should be initiated remains unanswered and prospective human studies have yet to define the ideal time to begin cooling patients [22].

Given the strong effect of therapeutic hypothermia with a protocol chosen on the basis of experimental data, there might be potential to further optimize the treatment and gain an even better neurologic outcome for patients after a global cerebral ischemic event [20,23]. At present, hypothermia is initiated mainly in-hospital and for arrests with cardiac etiology and a typical clinical presentation. However, there are no data to support whether earlier and faster cooling, already initiated in the out-of-hospital phase, may be better for preserving neurologic function [24-26]. Further studies should attempt to determine the time point at which the initiation of therapeutic hypothermia becomes most beneficial to patients who achieve restoration of spontaneous circulation following cardiac arrest [7,23]. Additional research is required to explore the best methods for induction of therapeutic hypothermia, including the most appropriate time and location of therapeutic hypothermia initiation and the optimal means to induce temperature reduction.

## Conclusions

In comatose cardiac arrest patients treated with therapeutic hypothermia after return of spontaneous circulation, a faster decline in body temperature to the 34°C target appears to predict a less favorable neurologic outcome. A faster decline in body temperature may simply indicate a more severe ischemic insult with resulting impairments in thermoregulation and thus predicts a worse outcome. Nevertheless, prospective studies should be conducted to confirm this observation and, in turn, address whether such a finding is simply a marker for a more severe insult or whether controlling the rate of cooling could favorably alter the outcome.

## Key messages

• In cardiac arrest managed with therapeutic hypothermia after resuscitation, reaching the 34°C target faster retrospectively predicts a poor neurologic outcome.

• A faster decline in body temperature may indicate a more severe ischemic insult with impaired thermoregulation and thus predicts a worse outcome.

• Prospective studies should address whether this finding is simply a marker for a more severe insult or whether controlling the rate of cooling can favorably alter outcome.

## Abbreviations

CPC: cerebral performance category; IQR: interquartile range.

## Competing interests

The authors declare that they have no competing interests.

## Authors' contributions

WB, HH, and MHo contributed to the steering and writing committees, to the conception and design, and to the analysis and interpretation of data. MHa contributed to the writing committee and to the acquisition, analysis, and interpretation of data. FS contributed to the steering and writing committees, to the conception and design, and to the acquisition, analysis, and interpretation of data. CT contributed to the writing committee and to the acquisition of data. MU contributed to the temperature data collection, to the writing committee, and to the acquisition and interpretation of data. All authors were involved in drafting the manuscript or revising it critically for important intellectual content. All authors read and approved the final manuscript.
